# A study of the impact of an interprofessional education module in Vietnam on students’ readiness and competencies

**DOI:** 10.1371/journal.pone.0296759

**Published:** 2024-02-14

**Authors:** Huyen Thi Thanh Nguyen, Johan Wens, Giannoula Tsakitzidis, Martin Valcke, Hoa Thi Nguyen, Tuan Quang Duong, Cuc Thi Nguyen, Dao Anh Hoang, Yen Thi Bach Hoang, Lan Thi Ngoc Duong, Hung Van Nguyen, Thanh Viet Truong, Huy Vu Quoc Nguyen, Tam Minh Nguyen

**Affiliations:** 1 Department of Family Medicine, Hue University of Medicine and Pharmacy, Hue University, Hue, Vietnam; 2 Department of Family Medicine and Population Health, Faculty of Medicine and Health Sciences, University of Antwerp, Antwerpen, Belgium; 3 Department of Educational Studies, Faculty of Psychology and Educational Sciences, Ghent University, Ghent, Belgium; 4 Faculty of Odonto-Stomatology, Hue University of Medicine and Pharmacy, Hue University, Hue, Vietnam; 5 Faculty of Public Health, Hue University of Medicine and Pharmacy, Hue University, Hue, Vietnam; 6 Faculty of Nursing, Hue University of Medicine and Pharmacy, Hue University, Hue, Vietnam; 7 Faculty of Vietnamese Traditional Medicine, Hue University of Medicine and Pharmacy, Hue University, Hue, Vietnam; 8 Faculty of Pharmacy, Hue University of Medicine and Pharmacy, Hue University, Hue, Vietnam; 9 Department of Obstetrics & Gynaecology, Hue University of Medicine and Pharmacy, Hue University, Hue, Vietnam; International Medical University, MALAYSIA

## Abstract

**Introduction:**

The literature puts forward a range of challenges of interprofessional education (IPE) related to its planning, initiation, implementation, and especially to IPE assessment. The present study aims to map changes in students’ readiness and interprofessional collaboration competence (IPCC) in implementing an innovative IPE module. Potential differences in impact related to the health education programs and IPCC scores resulting from self-, peer-, and tutor assessments will also be analysed.

**Methods:**

A pre-post design was adopted. The student’s readiness for interprofessional learning was assessed using the Readiness for Interprofessional Learning Scale, and the student’s IPCC score was calculated based on self-, peer-, and tutor assessments with the interprofessional collaborator assessment rubric.

**Results:**

Students’ mean post-test readiness scores and mean post-test IPCC scores were significantly higher than the total and subscales/domain pre-test scores (p<0.01). No significant within-subject differences were observed in students’ readiness total or subscale scores when comparing health educational programs. However, significant differences were observed in students’ mean total IPCC scores between programs (p<0.01). Significant differences in students’ average IPCC scores were found when comparing self-, peer- and tutor assessment scores in six domains (p<0.01). Also, significant correlations between peer and tutor assessment scores were observed (p<0.01).

**Conclusion:**

The IPE module, designed and implemented to focus on patient-centred practice within a primary care context, positively impacted students’ readiness and IPCC development. These results offer insights to expand the implementation of the IPE module to all health educational programs.

## Introduction

In an increasingly complex and changing healthcare environment, interprofessional collaboration (IPC) is crucial to improve patient safety and increase healthcare outcomes [1, 2]. Interprofessional education (IPE) is beneficial for preparing healthcare professionals to work collaboratively to provide patient-centred care [3, 4]. Since 2002, IPE has been defined by the Centre for the Advancement of Interprofessional Education Globally as what "occurs when students from two or more professions learn about, from and with each other to enable effective collaboration and improve health outcomes" [5]. IPE programs have been offered for undergraduate and postgraduate education in developed and developing countries, benefiting these countries and academic institutions [6]. Also, the benefits of IPE for education, practice, and even health policy in many countries have been reported [7]. IPE benefits for practice and policy of the healthcare systems have been indicated, including improved workplace practices, better health outcomes, improved quality of care for patients and patient safety, enhanced staff morale, staff retention, and better workplace productivity and health workforce recruitment [7]. However, in Vietnam, IPE training in universities remains limited, which leads to primary healthcare providers lacking IPC in clinical practice, especially in chronic disease management [8]. In this context, the University of Medicine and Pharmacy, Hue University (HueUMP), has developed an innovative IPE program for seven health education programs focusing on patient-centred care practice.

The implementation and evaluation of IPE modules have already been reported in the literature before [4, 9]. But simply copying and applying existing modules is not recommended. The design of an IPE module should be context-specific, meet the needs of a specific educational program and be required to serve all related programs/professions to result in the successful implementation of the IPE [10]. The latter is especially true when the IPE program has to fit different health education programs, has to build on local practice simulation cases, has to mirror local clinical practices, and be aligned with the local community setting to guarantee that students develop their interprofessional collaboration competence (IPCC) in a real-life practice focused on localised patient-centred care [11–13]. The above put forward the critical research problem of the present study: What is the impact of implementing an innovative localised competence-based IPE module in seven health education programs?

In medical education, competency-based education (CBE) was defined as an approach to preparing healthcare providers for practice that is fundamentally oriented to graduate outcome abilities and organised around competencies derived from an analysis of societal and patient needs [14]. CBE promises greater accountability, flexibility, and learner-centredness regardless of time-based training. CBE has become foundational for developing the IPE [15]. Studying the impact of the innovative IPE module requires adequate evaluation tools [16]. However, adopting a CBE approach in the context of the current study influences the choice of an assessment instrument. In line with CBE guidelines [17], teachers and learners share responsibilities in competence development [18, 19]. For instance, students adopt a self-reflective position and take responsibility for their learning self-assessment. Also, sharing with others is beneficial in CBE, as reflected in peer and teacher/tutor assessments, to receive feedback to guide their development and the acquisition of the necessary competencies [20]. In the context of this study, the resulting three assessment approaches ‐ self-, peer-, and tutor assessment–offer three different windows to study the development of students’ interprofessional competence development. This also offers an opportunity to explore the levels of agreement between the three assessment approaches.

In view of an empirical assessment of the impact of the module, an established assessment instrument referred to in the literature focuses on students’ readiness for interprofessional learning [21–23]. This four-dimensional assessment approach mirrors critical factors that play a role in interprofessional work, including (1) relationships between professional groups (values and beliefs), (2) collaboration and teamwork (knowledge and skills needed), (3) roles and responsibilities (what people do), and (4) benefits for patients, professional practice and personal growth (what actually happens)” [21].

Building on the above, the actual mastery of the IPCC can be assessed to track students’ development in their interprofessional learning [24, 25]. At the same time, the assessment approaches can potentially direct IPE’s (re)design in view of beneficial and intended outcomes [26].

## Materials and methods

### Aim

This study aimed to map students’ readiness and interprofessional collaboration competence (IPCC) changes by implementing an innovative IPE module. Potential differences in impact related to the health education programs and IPCC scores resulting from self-, peer-, and tutor assessments will also be analysed.

### Study design

A pre-post quasi-experimental design was adopted to determine students’ readiness and IPCC before and after the IPE module. According to Kirkpatrick’s model, four levels of training evaluation can be differentiated. Level one ’*reaction’* centres on how the delegates feel and react personally reactions to the training/learning experience; level two ’*learning*’ refers to knowledge development and the increase in intellectual capabilities building on the learning experiences; level three ’*behaviour*’ points at the application of what was learned and that results in behavioural changes; and level four ’*results’* points at the actual impact on the organisation, business or environment resulting from the better performance of the trainee [27]. The design aimed to assess learners at level three ‐ behaviour out of four levels of training evaluation based on Kirkpatrick’s model [27] and the IPE outcomes of Barr et al. [28]. Students’ readiness for interprofessional learning was assessed before the beginning and at the end of the IPE module. Additionally, students’ IPCC was determined during the beginning of the clinical practice session and at the end of the IPE module. Students’ IPCC was assessed parallel by self-, peer-, and tutor assessment using the same assessment tool.

### Study population and sample

Students were invited to voluntarily register for participation in this IPE module, including students in the fifth year in Medicine, Odonto-Stomatology, Preventive Medicine, and Vietnamese traditional medicine (VTM), the fourth year in Pharmacy, and the third year in Nursing, and the second year in Midwifery. This group of students were practising in the clinical rotations. All involved students have had the same lessons in basic medicine so far in their curriculum and have been trained similarly. These students acquired basic knowledge to develop a care plan for patients from the perspective of their profession. Additionally, because no IPE course was offered at HueUMP, students only attended IPE training after participating in this IPE module. The IPE module information, learning goals, and learning schedule were provided. Four hundred fifty-two students were registered to participate in the module. The recruitment was from 17th May 2022 to 22nd May 2022. Two hundred ten students were randomly chosen for the 30 places per education program. The 210 enrolled students were randomly divided into 30 interprofessional groups, each with seven students from seven different health education programs. All participants’ written informed consent was obtained before participating in the IPE module. All students who completed the IPE module were chosen to participate in this study.

### Instrument

The Readiness for Interprofessional Learning Scale (RIPLS) was used to assess students’ readiness for interprofessional learning [29]. The RIPLS consisted of 19 items divided into four subscales, including (1) ’teamwork and collaboration’ (items 1–9), (2) ’negative professional identity’ (items 10–12), (3) ’positive professional identity’ (items 13–16), and (4) ’roles and responsibilities’ (items 17–19). Respondents used a 5-point Likert scale to reply to statements (1 = strongly disagree, 5 = strongly agree). The total RIPLS score ranged from 19 to 95. The psychometric information about the RIPLS in the Vietnamese context was reported in a previous study at HueUMP, and the relative independence of the four subscales was pointed out [30].

The interprofessional collaborator assessment rubric (ICAR) was used to measure students’ interprofessional competencies performance [31]. The ICAR was proven to assess knowledge/skills and behavioural acquisition [31]. This tool was also used for self-assessment (students evaluated by themselves), peer assessment (students assessed by a peer), and tutor assessment (students evaluated by a tutor) as individual assessments in groups. ICAR contained 31 dimensions organised into six domains, consisting of (1) communication, (2) collaboration, (3) roles and responsibilities, (4) collaborative patient/client-family-centred approach, (5) team functioning, and (6) conflict management/resolution. For each dimension, students’ performance was assessed by a scale of four levels (1 = minimal, 2 = developing, 3 = competent, 4 = mastery). The assessment was implemented by what was appropriate to the context/task. These four levels were commensurate with the frequency of performing their ability, including ’never’, ’occasionally’, ’frequently’, and ’consistently’. The reliability and validity of the ICAR have been assessed in different cultural contexts, including in Canada [32, 33], in Iran [34], in Indonesia [35], and in Sweden [36]. However, psychometric information about the ICAR in the Vietnamese context is unavailable. Preliminary analyses will be carried out to document instrument quality.

The RIPLS and ICAR were translated into Vietnamese using the back translation method to ensure consistency between these instruments’ original and translated versions [37]. Permission to translate and use these instruments was obtained.

### Pilot study

The IPE module was designed as a CBE learning experience spread over ten sessions, including two theory sessions and eight practice-based approach sessions. Multiple interactive learning activities were designed to create opportunities for students to apply their uni-professional competencies within an interprofessional setting. The IPE was developed as a blended learning module combining online and face-to-face learning. All learning materials were available for students to access through an online platform of the Learning Management System. Students received the assignment, submitted their work and received feedback from tutors via the Learning Management System. Six workshops were organised for faculty development to train all 35 tutors before starting the IPE module. Six faculty development workshops were organised to train all 35 tutors before starting the IPE module. These workshops were coordinated by experts from Ghent University, University of Antwerp, University of Liege, Belgium and Harvard University, USA. The content of these workshops focussed on introducing the conceptual base of IPE, the scope of IPE affecting each profession in a team, how to develop a teaching plan, building interprofessional case studies, designing assessment strategies and tips for guiding interprofessional student teams. All tutors and students were also trained in using the ICAR tool.

The pilot study on the IPE module was implemented from 28th May 2022 to 30th July 2022. One IPE session was implemented per week (see S1 Table). The first session was organised while introducing IPE in a plenary session, getting to know each other and setting up team-building exercises. Students started working in stable groups (N 7) throughout the IPE module. In session two, students tackled four case studies through small group discussions. In the third session, students practised IPE via a simulation involving a standardised patient; each interprofessional student team communicated with the standardised patient and collaborated to develop an interprofessional care plan for the patient. Session four focused on involvement in clinical practice in health facilities at the primary care level. Students communicated with actual patients with chronic conditions and worked in their teams to design care plans for these patients. In the fifth session, students visited patients at home to explore the patient’s family situation. This was expected to help focus on a comprehensive care plan. Session six was organised to tackle a multiprofessional debriefing. Student groups presented their care plans to other groups and tutors representing different professions. They received feedback and were involved in self-reflection activities. Sessions four, five, and six were reiterated in sessions seven, eight and nine. This allowed students to follow up with their patients with chronic conditions and could improve their ideas and resulting performance. In session ten, students’ IPCC assessment was organised.

A shared feature of the sessions was the focus on patient-centred practice in which students from different programs collaborated to develop care plans for patients with morbidity health conditions in a primary care context. The design and implementation of the IPE module have been described in more detail in another paper that will be published soon.

### Data collection

A survey to determine students’ readiness for interprofessional learning was conducted with the pre-test immediately before starting the module and the post-test at the end of the module. The RIPLS was used. The data collection was held in classrooms. Students were informed about the aim of the study and about how to complete the tool before data collection. Written informed consent was obtained from participants. The researchers were present to answer any questions during the data collection. Students’ Demographic information was collected, including sex, age, health education program, current year of study, and grade point average (GPA) in a previous academic year.

Two Objective Structured Clinical Examination moments (OSCEs) were organised to assess students’ IPCC using the ICAR: one was at the beginning of the clinical sessions for the pre-test, and one was at the end of the module for the post-test. The OSCE was organised as a clinical simulation involving standardised patients. The same assessment base was offered for all groups with the same case study scenario. Each group was in a different room, such as a simulated examination room or a classroom. The OSCE was processed in four steps, including (1) students receiving a paper case with the patient’s basic information and preparing, (2) taking a medical history of a standardised patient, (3) collaborating within the team to make a care plan for the patient, (4) doing a consultation with the standardised patient to provide their care plan. During the OSCE, each tutor observed and evaluated all students in a group. At the end of an OSCE, a reflection session was organised where tutors and students gave feedback to each other in each group. The OSCE activity was videotaped to help tutors check their evaluation and scoring by themselves or other tutors. Therefore, the reliability of this evaluation could also be checked or tested. After finishing the OSCEs, students assessed themselves and others in their group via a Google Form of the ICAR.

### Ethics

The study protocol was approved by the Medical Ethics Committee of the University of Medicine and Pharmacy, Hue University [number: H2022/003 10th January 2022]. All methods were performed in accordance with the relevant guidelines and regulations.

### Quality of the Vietnamese version of the ICAR

The internal consistency of the ICAR in self-, peer-, and tutor-assessment ranged from 0.92 to 0.96. Cronbach alpha values for the subscales were good in communication, collaboration, roles and responsibilities, collaborative patient/client-family-centred approach, and TP and lowest for the conflict management/resolution subscale (α = 0.60) in the pre-test of tutor assessment. Pearson’s correlations revealed highly significant correlations between the six subscales in self-assessment (between *r* = 0.887 and *r* = 0.442), peer assessment (between *r* = 0.914 and *r* = 0.562), tutor assessment (between *r* = 0.888 and *r* = 0.475), pointing to the relative independence of the six subscales. Pearson’s correlations pointed to significant correlations between the tutor assessment and peer assessment in the five domains in the pre-test, including communication (r = 0.302), collaboration (r = 0.293), roles and responsibilities (r = 0.161), collaborative patient/client-family-centred approach (r = 0.263), and team functioning (r = 0.228) (Table 1). In the post-test, significant Pearson correlations were found in the domain of communication (r = 0.189), collaboration (0.231), and roles and responsibilities (*r* = 0.149). No significant correlations were found in self-assessment with peer- and tutor assessment.

**Table 1:** Pearson correlation between self-, peer-, and tutor-assessment.

	Tutor assessment ‐ Self-assessment		Tutor assessment ‐ Peer assessment		Self-assessment ‐ Peer assessment	
	Pre-test	Post-test	Pre-test	Post-test	Pre-test	Post-test
Communication	-0.034	0.133	0.302**	0.189**	0.015	0.01
Collaboration	-0.125	0.075	0.293**	0.231**	-0.070	0.046
Roles and responsibility	-0.091	0.103	0.161*	0.149*	0.007	0.033
Collaborative Patient/Client- Family Centred Approach	-0.052	0.029	0.263**	0.140	0.070	-0.041
Team Functioning	-0.016	0.133	0.228**	0.102	0.029	0.084
Conflict Management/ Resolution	-0.027	-0.001	0.020	0.110	0.076	-0.034

**p<0.01

*p<0.05

### Data analysis

Statistical Package for the Social Sciences (SPSS, version 28.0) was used for data analysis. The results were considered statistically significant if *p* <0.01. In the RIPLS, the coding was reversed for the ’negative professional identity’ and ’roles and responsibilities’ subscales to guarantee that higher scores reflect that students are more ready for interprofessional learning in the four subscales [30]. The preliminary analysis focused on the quality of the instrument (scale statistics and factor analysis). Descriptive statistics were also determined, and basic comparisons were conducted based on the demographic variables. The (sub)scale scores in RIPLS and ICAR were not normally distributed (Kolmogorov-Smirnov test p<0.05).

The mean RIPLS and ICAR scores were compared between pre-test and post-test and within the different health education programs. Based on the valid RIPLS and ICAR, the Wilcoxon Sign Rank test was performed to analyse pre-test and post-test differences. The Kruskal-Wallis H test was used to compare the RIPLS scores for different health education programs. A Bonferroni correction for significance was applied when comparing multiple groups to see which groups differed. The Friedman test compared the ICAR scores between self-, peer- and tutor assessments.

Missing data, which means the items were not observed or applicable, ranged from 54.7% to 100% in tutor-assessment items 7, 15, 16, 29, and 31. These dimensions were relevant to communication with individuals with impairments, responses to the failure of collaborative goals, and conflict, which rarely occur in an OSCE context. Therefore, these five items were removed from the ICAR tool for analysis. For the remaining 26 items, missing data ranged from 0% to 2.6% in self-assessment, from 0% to 7.9% in peer assessment, and from 0% to 22.6% in tutor assessment. All missing data in quantitative variables were replaced by imputed individual missing values from the data set of the item mean using Python pandas [38]. This means that missing scores were replaced with the individual’s average item score.

## Results

Out of 210 enrolled students, 190 students completed the IPE module and the questionnaires, representing an overall response rate of 90.5%. Student dropout was registered and seemed unrelated to the nature of the study. These students dropped out of the program together.

The demographic characteristics of students included in the study are summarised in Table 2. The number of students from different programs was almost similar, including 29 from medicine, 26 from nursing, 29 from pharmacy, 28 from preventive medicine, 27 from VTM, 24 from midwifery, and 27 from odonto-stomatology. There were 18.9% male students (n = 36) and 81.1% female students (n = 154) in the total sample. The rate of female students was highest in midwifery (100%) and nursing (96.2%). The average age of the students was 22.20 (±1.24). The mean GPAs of students in the previous academic year were mainly ‘good’ (56.8%) and ‘very good’ (30.5%). The rate of ‘excellent’ and ‘average’ students was similar, with 6.3%. There was no below-average student.

**Table 2:** Demographic characteristics of participants (N 190).

		Total population	Medicine	Nursing	Pharmacy	Preventive Medicine	Vietnamese traditional medicine	Midwifery	Odonto-Stomatology
**Sex [n (%)]**									
	Male	36 (18.9)	12 (41.4)	1 (3.8)	4 (13.8)	5 (17.9)	4 (14.8)	0 (0.0)	10 (37.0)
	Female	154 (81.1)	17 (58.6)	25 (96.2)	25 (86.2)	23 (82.1)	23 (85.2)	24 (100.0)	17 (63.0)
	Total	190 (100.0)	29 (100.0)	26 (100.0)	29 (100.0)	28 (100.0)	27 (100.0)	24 (100.0)	27 (100.0)
**Age [mean (SD)]**									
		22.20 (1.24)	23.03 (0.33)	20.77 (0.43)	21.97 (0.33)	23.18 (0.48)	23.15 (0.46)	20.04 (0.69)	22.89 (0.42)
**GPAs in the previous academic year [n (%)]**									
	Excellent (3.6–4.0)	12 (6.3)	4 (15.4)	0 (0.0)	1 (3.4)	1 (3.6)	5 (18.5)	0 (0.0)	1 (3.7)
	Very good (3.2–3.6)	58 (30.5)	12 (41.4)	3 (11.5)	6 (20.7)	10 (35.7)	16 (59.3)	1 (4.2)	9 (33.3)
	Good (2.5–3.2)	108 (56.8)	13 (44.8)	22 (84.6)	21 (72.4)	17 (60.7)	6 (22.2)	13 (54.2)	17 (63.0)
	Average (2.0–2.5)	12 (6.3)	0 (0.0)	1 (3.8)	1 (3.4)	0 (0.0)	0 (0.0)	10 (41.7)	0 (0.0)

Table 3 displays the mean RIPLS scores resulting from the comparisons of pre-test and post-test and the multiple comparisons between programs. The overall mean RIPLS score was 75.49 (SD± 8.82) in the pre-test and 82.97 (SD± 6.87) in the post-test (max = 95). In total population, the Wilcoxon Sign Rank test indicated that students’ mean RIPLS scores in the post-test were significantly higher than in the pre-test (p<0.001) in the total RIPLS score and four subscales. Looking at programs, higher significant total RIPLS mean scores were found in all programs. However, in the four subscales, no significant differences were found in nursing and VTM in the ’teamwork and collaboration’ and ’positive professional identity’ subscales, in pharmacy, VTM, midwifery in the ’negative professional identity’ subscale, and midwifery in the ’roles and responsibilities’ subscale. The Kruskal-Wallis H test results revealed no significant differences observed in students’ RIPLS scores between programs in the total and four subscales in both the pre-test and the post-test.

**Table 3:** Multiple comparisons of students’ mean RIPLS scores between pre-test and post-test and between programs.

		Total population [X_mean_±SD]	Medicine [X_mean_±SD]	Nursing [X_mean_±SD]	Pharmacy [X_mean_±SD]	Preventive Medicine [X_mean_±SD]	Vietnamese traditional medicine [X_mean_±SD]	Midwifery [X_mean_±SD]	Odonto-Stomatology [X_mean_±SD]	p-value
		n = 190	n = 29	n = 26	n = 29	n = 28	n = 27	n = 24	n = 27	
**Total RIPLS score**										
	Pre-test	75.49±8.82	75.45±6.95	77.69±13.49	76.10±6.84	72.89±7.78	77.70±6.08	75.24±9.39	75.49±9.22	0.078
	Post-test	82.97±5.87	81.72±5.76	84.27±5.13	83.24±6.60	82.11±6.12	82.67±5.68	81.96±5.34	84.89±6.06	0.356
**p-value**		**<0.001**	**<0.001**	**0.002**	**<0.001**	**<0.001**	**<0.001**	**0.003**	**<0.001**	
**Teamwork and collaboration**										
	Pre-test	37.62±4.62	37.59±3.36	38.31±6.93	37.45±3.49	36.43±3.88	39.04±3.48	37.50±4.75	37.11±5.59	0.131
	Post-test	40.65±2.92	39.93±2.98	41.08±2.62	40.93±2.80	40.32±3.03	40.37±3.01	40.25±2.74	41.67±3.16	0.327
**p-value**		**<0.001**	**<0.001**	0.064	**<0.001**	**<0.001**	0.062	**0.014**	**<0.001**	
**Negative professional identity**										
	Pre-test	11.95±2.43	12.21±2.34	12.38±2.53	12.55±2.01	11.54±2.25	12.00±2.39	11.58±2.99	11.33±2.53	0.446
	Post-test	13.26±1.72	13.31±1.48	13.54±1.21	13.17±2.45	13.32±1.68	13.00±1.33	13.13±2.36	13.37±1.24	0.824
**p-value**		**<0.001**	**0.005**	**0.012**	0.072	**<0.001**	0.036	0.041	**<0.001**	
**Positive professional identity**										
	Pre-test	16.51±2.23	16.31±1.77	16.77±3.29	16.34±2.19	16.18±2.06	17.56±1.95	16.67±2.04	15.81±1.86	0.057
	Post-test	17.79±1.64	17.31±1.56	17.92±1.49	18.03±1.66	17.71±1.92	17.89±1.48	17.88±1.51	17.81±1.82	0.807
**p-value**		**<0.001**	**0.007**	0.034	**<0.001**	**0.003**	0.391	**0.009**	**<0.001**	
**Roles and responsibilities**										
	Pre-test	9.41±1.80	9.34±2.06	10.23±2.16	9.76±1.21	8.75±1.27	9.11±1.83	9.50±2.28	9.19±1.36	0.095
	Post-test	11.27±1.87	11.17±1.79	11.73±1.54	11.10±2.34	10.75±2.24	11.41±1.78	10.71±1.37	12.04±1.48	0.090
**p-value**		**<0.001**	**0.002**	**<0.001**	**<0.001**	**<0.001**	**<0.001**	0.043	**<0.001**	

Table 4 shows the mean and standard deviation values for the mean total ICAR scores in the total population and different programs. The Wilcoxon Sign Rank test indicated significantly higher students’ mean total ICAR scores in the post-test compared to those in the pre-test in the total population and all programs (p<0.001), except midwifery in self-assessment and medicine in peer assessment. The significant differences in the mean total ICAR scores of students between programs in the post-test (p = 0.005) of self-assessment, in the pre-test (p = <0.001) of peer assessment, and the pre-test (p<0.001) of tutor assessment were found using The Kruskal-Wallis H test. Multiple pairwise comparison analysis was carried out, applying Bonferroni correction for significance. This clarified that in the post-test of self-assessment, preventive medicine students’ mean total ICAR scores were significantly higher than midwifery students (p = 0.005). Students’ mean total ICAR scores in medicine were significantly higher than in midwifery (p<0.001) in the pre-test of peer assessment. Looking at the pre-test of tutor assessment, medical students had significantly higher mean total ICAR scores than students in midwifery (p<0.001), pharmacy (p<0.001), preventive medicine (p = 0.008), and nursing (p = 0.009). Additionally, students’ mean total ICAR scores in Odonto-Stomatology were significantly higher than in midwifery (p = 0.005), and VTM students were significantly higher than midwifery students (p = 0.008).

**Table 4:** Multiple comparisons of students’ mean total ICAR scores between pre-test and post-test and between programs.

		Total population [X_mean_±SD]	Medicine [X_mean_±SD]	Nursing [X_mean_±SD]	Pharmacy [X_mean_±SD]	Preventive Medicine [X_mean_±SD]	Vietnamese traditional medicine [X_mean_±SD]	Midwifery [X_mean_±SD]	Odonto-Stomatology [X_mean_±SD]	p-value
		n = 190	n = 29	n = 26	n = 29	n = 28	n = 27	n = 24	n = 27	
**Self-assessment**										
	Pre-test	2.99±0.44	2.89±0.35	2.97±0.50	2.98±0.45	3.17±0.43	2.96±0.38	2.95±0.53	3.00±0.40	0.340
	Post-test	3.33±0.39	3.37±0.38	3.31±0.37	3.26±0.35	3.53±0.36	3.40±0.33	3.08±0.46	3.31±0.39	**0.005**
	p-value	**<0.001**	**<0.001**	**0.006**	**0.002**	**<0.001**	**<0.001**	0.134	**0.002**	
**Peer assessment**										
	Pre-test	2.95±0.47	3.29±0.48	2.93±0.49	2.94±0.43	2.90±0.40	3.00±0.46	2.66±0.42	2.88±0.40	**<0.001**
	Post-test	3.40±0.41	3.55±0.48	3.31±0.37	3.38±0.46	3.41±0.35	3.31±0.29	3.27±0.45	3.57±0.37	0.018
	p-value	**<0.001**	0.050	**0.008**	**<0.001**	**<0.001**	**0.006**	**<0.001**	**<0.001**	
**Tutor assessment**										
	Pre-test	1.80±0.34	2.08±0.23	1.76±0.32	1.69±0.27	1.80±0.32	1.84±0.31	1.49±0.30	1.86±0.33	**<0.001**
	Post-test	3.00±0.42	3.20±0.40	2.98±0.38	2.97±0.46	3.00±0.37	3.04±0.42	2.72±0.39	3.01±0.44	0.012
	p-value	**<0.001**	0.050	**0.008**	**<0.001**	**<0.001**	**0.006**	**<0.001**	**<0.001**	

Table 5 summarises the mean and standard deviation values for the mean ICAR score in six domains in different assessment methods. Looking at the differences in students’ mean ICAR scores between self-, peer- and tutor assessments, the Friedman test results point to significant differences in six domains in all the pre-test and the post-test (p<0.001) (Table 5). In pairwise comparison analysis, after the Bonferroni correction, the mean ICAR scores were significantly higher in self-assessment than in tutor assessment (p<0.001). Also, students’ mean ICAR scores in peer assessment were significantly higher than in tutor assessment (p<0.001). The Wilcoxon Sign Rank test was also performed, and students’ mean ICAR scores in the post-test were significantly higher than in the pre-test (p<0.001) in six domains in all self-, peer-, and tutor-assessments.

**Table 5:** Comparison of students’ mean ICAR scores between self-, peer-, and tutor-assessments.

Domain	Pre-test				Post-test			
	Self- [X_mean_±SD]	Peer- [X_mean_±SD]	Tutor- [X_mean_±SD]	p-value	Self- [X_mean_±SD]	Peer- [X_mean_±SD]	Tutor- [X_mean_±SD]	p-value
Communication	2.87±0.52	2.86±0.51	1.85±0.40	**<0.001**	3.25±0.46	3.36±0.50	3.04±0.49	**<0.001**
Collaboration	2.98±0.52	2.92±0.56	1.66±0.39	**<0.001**	3.35±0.46	3.34±0.47	2.88±0.50	**<0.001**
Roles and responsibility	2.76±0.55	2.85±0.55	1.61±0.35	**<0.001**	3.16±0.41	3.39±0.46	2.82±0.48	**<0.001**
Collaborative Patient/Client- Family Centred Approach	2.95±0.54	2.88±0.57	1.66±0.44	**<0.001**	3.30±0.49	3.37±0.43	2.93±0.51	**<0.001**
Team Functioning	3.07±0.50	3.06±0.50	1.89±0.36	**<0.001**	3.39±0.46	3.47±0.43	3.06±0.46	**<0.001**
Conflict Management/ Resolution	3.30±0.51	3.14±0.48	2.11±0.51	**<0.001**	3.52±0.48	3.48±0.50	3.24±0.55	**<0.001**

## Discussion

In line with CBE, the IPE module was designed so that students can learn, apply and practice the IPE knowledge, skills and attitudes within the context of simulation with standardised patients, clinical practice, and community-based education. This study aimed to determine the impact of the IPE module on students’ readiness and IPCC and to study differences between health education programs. The difference in students’ IPCC scores between self-, peer-, and tutor assessments was explored.

In this study, the high response rate of students provided a high confidence level to arrive at robust results. Students in each health educational program had a similar participation rate. Students’ GPAs in the previous academic year were a standard distribution and similar between programs. This supports the representation of the population in all health educational programs [39].

The weaknesses of the RIPLS in the reliability of measurements by the subscales have been indicated [23, 40]. The low reliability in the ’roles and responsibilities’ subscale of the RIPLS in the Vietnamese context was also reported and analysed in a previous study at HueUMP [30]. The reliability analysis pointed to the particular nature of this scale. In the three items in the ’roles and responsibilities’ subscale, three roles represented different valid sets of responsibilities in a professional. The focus on the different roles/responsibilities affected its reliability but underpinned the content validity of this subscale [30].

The finding showed statistically significant improvements in students’ readiness, as measured by the RIPLS, in the total RIPLS score and four subscales. Working in a stable interprofessional student team throughout the module, students had an experience of collaborating with other professionals in different scenarios. This has been designed and implemented in the flowing learning activities, including team-building, group discussion on case studies, practice in simulation with standardised patients, clinical practice, and home visits. That made students approach and experience step-by-step with the actual IPC. That promoted students’ autonomous motivation and improved their readiness for interprofessional learning. Similar results were found in several studies that students’ perceptions of IPC and clinical decision-making could be enhanced by IPE [41–43]. Also, Reilly et al. indicated that IPE training in community-based practice positively influences students’ attitudes and understanding toward IPC [44]. However, looking at each subscale, no significant increases were found in nursing and VTM in the ’teamwork and collaboration’ and ’positive professional identity’ subscales, in pharmacy, VTM, and midwifery in the ’negative professional identity’ subscale, and midwifery in the ’roles and responsibilities’ subscale. This could be explained by the higher readiness of participating students for interprofessional learning at the beginning of the IPE module compared to the general groups in the previous study [30]. The voluntary participating students may also influence this.

A statistically significant increase in students’ IPCC, measured by the ICAR total scores, was also found in all programs in self-, peer-, and tutor assessments in all six domains. This suggests that the IPE module positively impacted all participating health educational programs. Moreover, in the post-test, all six domains in self- and peer assessments and three in tutor assessment, including communication, team functioning and conflict management/resolution, had mean scores above three out of four. That means students’ IPCC reached the level of “competent” (score = 3) compared to the level of “developing" (score = 2) and "minimal" (score = 1) in the pre-test. This finding demonstrated that the impact is also positive in improving students’ IPCC through the IPE module. This result suggests that the IPE module was well-designed in an appropriate primary care context and improved the IPCC of all relevant health educational programs. The primary care setting is a good practice context for IPE where undergraduate students from different programs have optimal opportunities to collaborate to practice patient-centred care as close to real-life situations as possible [45, 46]. Similar to this study, Miselis et al. showed that a longitudinal experiential IPE program was valued in addressing key IPCC in the clinical learning environment in the primary care setting in the USA [47]. In addition, a strong effect of IPE community-based learning on improving students’ IPCC, measured by the ICAR, was indicated in Indonesia [35].

However, there were no significant improvements in medical students’ IPCC in peer- and tutor assessment. This could be explained by the higher ICAR scores of medical students given by peers and tutors with higher expectations at the pre-test than students in other programs. Moreover, medical students may know better about the roles and leadership than other students. Also, preventive medicine students gave themselves higher ICAR scores compared to midwifery students in the post-test, but their peers and tutors did not give them higher scores than others. At the end of the IPE module, the IPCC scores of students showed no significant difference in all programs. In contrast to our finding, the mean ICAR scores of medical students were higher than midwifery students [35]. However, similar results were revealed in the study by Mark et al., who found that IPCC scores of students from different professions did not significantly differ [32]. This is a good impact of the IPE programs on the equal demonstration of IPCC, equal participation and contributions among programs/professionals [48].

Assessed by different actors, the analysis focuses on students’ IPCC scores in self-, peer-, and tutor assessments. The results showed that tutors gave a lower ICAR score for students than their peers and students themself. Students usually overestimate their performance [49]. In addition, correlations were found in peer- and tutor assessments in five domains, including communication, collaboration, roles and responsibilities, collaborative patient/client-family-centred approach, and team functioning. Self-assessment was different from the two other assessments. The correlation between peer- and tutor assessment was also revealed in the research by Papinczak et al. [50]. However, Alias et al. indicated a different finding that self- and peer assessment correlated with each other and did not correlate with teacher assessment of team-working skills [49]. This lack of correlation can be explained by the different abilities to use the assessment tool, and the IPCC of students would score differently [51]. However, by doing peer assessment, students better reflect on their work, supporting the development of student’s skills in reflection and self-awareness and enhancing students’ professional cooperation by improving their communication and attitudes toward active participation [52]. As a competency-based assessment method, the self-, peer-, and tutor assessments were used in the IPE module to reduce individual bias and consisted of analysis from different points of view [53]. These results also supported the inclusion of competency-based assessment scores in deciding students’ overall grades. Nevertheless, assessing IPE is challenging, with the involved struggles appearing to be grounded in limited resources for IPE assessment and logistical challenges with organising assessments for many students [54]. This needs to be investigated in further study.

A limitation of this study is related to comparing midwifery to students from other programs because of the less experience in clinical practice and professional identity of midwifery students. Because the health educational program for midwifery was only implemented in two years, there was no higher year of midwifery students. In the design of the IPE module, second-year midwifery considered that they can learn IPE within all learning activities to involve programs in our IPE module.

## Conclusions

The IPE module, designed and implemented to focus on patient-centred practice within a primary care context, positively impacted the improvement of students’ readiness and IPCC in the total and all subscales/domains. The IPE module also strongly impacted the readiness and IPCC of students from all participating health educational programs. A competency-based method using self-, peer-, and tutor assessment is recommended to evaluate students’ IPCC to optimise the quality of teaching and learning. These results offer insights into adapting the IPE module throughout the curriculum and expanding the IPE module to all health educational programs.

## Supporting information

S1 Table Learning activities of the interprofessional education module. (DOCX)

S1 Dataset Interprofessional collaboration competencies dataset. (SAV)

## References

[1] Reeves S, Perrier L, Goldman J, Freeth D, Zwarenstein M. Interprofessional education: effects on professional practice and healthcare outcomes. Cochrane Database of systematic reviews. 2013;(3). Epub 2013/04/02. doi: 10.1002/14651858.CD002213.pub3 ; PubMed Central PMCID: PMC6513239. 23543515 PMC6513239

[2] Zwarenstein M, Goldman J, Reeves S. Interprofessional collaboration: effects of practice‐based interventions on professional practice and healthcare outcomes. Cochrane database of systematic reviews. 2009;(3). doi: 10.1002/14651858.CD000072.pub2 19588316

[3] WHO. Framework for action on interprofessional education and collaborative practice: World Health Organization; 2010. 21174039

[4] Guraya SY, Barr H. The effectiveness of interprofessional education in healthcare: A systematic review and meta-analysis. The Kaohsiung journal of medical sciences. 2018;34(3):160–5. doi: 10.1016/j.kjms.2017.12.009 29475463 PMC12977169

[5] CAIPE. Interprofessional education: today, yesterday and tomorrow: a review.: Fareham UK: CAIPE; 2002. Available from: https://www.caipe.org/resources/publications/caipe-publications/caipe-2002-interprofessional-education-today-yesterday-tomorrow-barr-h.

[6] Herath C, Zhou Y, Gan Y, Nakandawire N, Gong Y, Lu Z. A comparative study of interprofessional education in global health care: a systematic review. Medicine. 2017;96(38). Epub 2017/09/21. doi: 10.1097/MD.0000000000007336 ; PubMed Central PMCID: PMC5617683. 28930816 PMC5617683

[7] Rodger S J. Hoffman S, Education WHOSGoI, Practice C. Where in the world is interprofessional education? A global environmental scan. Journal of interprofessional care. 2010;24(5):479–91. doi: 10.3109/13561821003721329 20718594

[8] Huyen NTT, Tsakitzidis G, Tam NM, Valcke M, Chuong HV, Wens J. Perceptions and experiences of primary healthcare providers toward interprofessional collaboration in chronic disease management in Hue, Vietnam. Journal of Interprofessional Care. 2023:1–10. Epub 2023/06/27. doi: 10.1080/13561820.2023.2227650 . 37366565

[9] Aldriwesh MG, Alyousif SM, Alharbi NS. Undergraduate-level teaching and learning approaches for interprofessional education in the health professions: a systematic review. BMC Medical Education. 2022;22:1–14. Epub 2022/01/05. doi: 10.1186/s12909-021-03073-0 ; PubMed Central PMCID: PMC8725543. 34980083 PMC8725543

[10] Bogossian F, New K, George K, Barr N, Dodd N, Hamilton AL, et al. The implementation of interprofessional education: a scoping review. Advances in Health Sciences Education. 2023;28(1):243–77. Epub 2022/06/11. doi: 10.1007/s10459-022-10128-4 ; PubMed Central PMCID: PMC9186481. 35689133 PMC9186481

[11] Gilbert JH. Interprofessional education for collaborative, patient-centred practice. Nursing leadership. 2005;18(2):32–8. doi: 10.12927/cjnl.2005.17181 16045054

[12] Herbert CP. Changing the culture: Interprofessional education for collaborative patient-centred practice in Canada. 2005;19(sup1):1–4. Epub 2005/08/13. doi: 10.1080/13561820500081539 . 16096140

[13] Baker C, Pulling C, McGraw R, Dagnone JD, Hopkins‐Rosseel D, Medves J. Simulation in interprofessional education for patient‐centred collaborative care. Journal of advanced nursing. 2008;64(4):372–9. doi: 10.1111/j.1365-2648.2008.04798.x 18764851

[14] Frank JR, Mungroo R, Ahmad Y, Wang M, De Rossi S, Horsley T. Toward a definition of competency-based education in medicine: a systematic review of published definitions. Medical teacher. 2010;32(8):631–7. Epub 2010/07/29. doi: 10.3109/0142159X.2010.500898 . 20662573

[15] Barr H. Competent to collaborate: towards a competency-based model for interprofessional education. Journal of interprofessional care. 1998;12(2):181–7. doi: 10.3109/13561829809014104

[16] Sunguya BF, Hinthong W, Jimba M, Yasuoka J. Interprofessional education for whom?—challenges and lessons learned from its implementation in developed countries and their application to developing countries: a systematic review. PLoS One. 2014;9(5):e96724. Epub 2014/05/09. doi: 10.1371/journal.pone.0096724 ; PubMed Central PMCID: PMC4014542. 24809509 PMC4014542

[17] Wagner SJ, Reeves S. Milestones and entrustable professional activities: the key to practically translating competencies for interprofessional education? Journal of interprofessional care. 2015;29(5):507–8. Epub 2015/06/11. doi: 10.3109/13561820.2014.1003636 . 26062110

[18] Holmboe ES, Sherbino J, Long DM, Swing SR, Frank JR, Collaborators IC. The role of assessment in competency-based medical education. Medical teacher. 2010;32(8):676–82. Epub 2010/07/29. doi: 10.3109/0142159X.2010.500704 . 20662580

[19] Burke J. Competency based education and training: Psychology Press; 1989.

[20] Bing-You RG, Trowbridge RL. Why medical educators may be failing at feedback. Jama. 2009;302(12):1330–1. doi: 10.1001/jama.2009.1393 19773569

[21] Parsell G, Bligh J. The development of a questionnaire to assess the readiness of health care students for interprofessional learning (RIPLS). Medical education. 1999;33(2):95–100. Epub 1999/04/22. doi: 10.1046/j.1365-2923.1999.00298.x . 10211258

[22] Lestari E, Stalmeijer RE, Widyandana D, Scherpbier A. Understanding students’ readiness for interprofessional learning in an Asian context: a mixed-methods study. BMC medical education. 2016;16:1–11. Epub 2016/07/17. doi: 10.1186/s12909-016-0704-3 ; PubMed Central PMCID: PMC4946087. 27422207 PMC4946087

[23] McFadyen AK, Webster VS, Maclaren W. The test-retest reliability of a revised version of the Readiness for Interprofessional Learning Scale (RIPLS). Journal of interprofessional care. 2006;20(6):633–9. Epub 2006/11/11. doi: 10.1080/13561820600991181 . 17095441

[24] Havyer RD, Nelson DR, Wingo MT, Comfere NI, Halvorsen AJ, McDonald FS, et al. Addressing the interprofessional collaboration competencies of the Association of American Medical Colleges: a systematic review of assessment instruments in undergraduate medical education. Academic Medicine. 2016;91(6):865–88. Epub 2015/12/26. doi: 10.1097/ACM.0000000000001053 . 26703415

[25] Smeets HWH, Sluijsmans DM, Moser A, van Merriënboer JJ. Design guidelines for assessing students’ interprofessional competencies in healthcare education: a consensus study. Perspectives on Medical Education. 2022;11(6):316–24. Epub 2022/10/13. doi: 10.1007/s40037-022-00728-6 ; PubMed Central PMCID: PMC9743853. 36223031 PMC9743853

[26] Curran VR, Sharpe D, Flynn K, Button P. A longitudinal study of the effect of an interprofessional education curriculum on student satisfaction and attitudes towards interprofessional teamwork and education. Journal of interprofessional care. 2010;24(1):41–52. Epub 2009/08/26. doi: 10.3109/13561820903011927 . 19705318

[27] Kirkpatrick JD, Kirkpatrick WK. Kirkpatrick’s four levels of training evaluation: Association for Talent Development; 2016.

[28] Barr H, Freeth D, Hammick M, Koppel I, Reeves S. Evaluations of interprofessional education: a United Kingdom review for health and social care: The United Kingdom Centre for the Advancement of Interprofessional Education with The British Educational Research Association.; 2000.

[29] McFadyen AK, Webster V, Strachan K, Figgins E, Brown H, McKechnie J. The Readiness for Interprofessional Learning Scale: a possible more stable sub-scale model for the original version of RIPLS. Journal of interprofessional care. 2005;19(6):595–603. Epub 2005/12/24. doi: 10.1080/13561820500430157 . 16373215

[30] Huyen NTT, Tam NM, Wens J, Tsakitzidis G, Len CTL, Van Chuong H, et al. Comparison of students’ readiness from six health education programs for interprofessional learning in Vietnam: A cross-sectional study. BMC Medical Education. 2023;23(1):798. Epub 2023/10/26. doi: 10.1186/s12909-023-04776-2 ; PubMed Central PMCID: PMC10601104. 37880693 PMC10601104

[31] Oates M, Davidson M. A critical appraisal of instruments to measure outcomes of interprofessional education. Medical Education. 2015;49(4):386–98. Epub 2015/03/25. doi: 10.1111/medu.12681 . 25800299

[32] Hayward MF, Curran V, Curtis B, Schulz H, Murphy S. Reliability of the Interprofessional Collaborator Assessment Rubric (ICAR) in Multi Source Feedback (MSF) with post-graduate medical residents. BMC Medical Education. 2014;14(1):1049. Epub 2015/01/01. doi: 10.1186/s12909-014-0279-9 ; PubMed Central PMCID: PMC4318203. 25551678 PMC4318203

[33] Curran V, Hollett A, Casimiro LM, Mccarthy P, Banfield V, Hall P, et al. Development and validation of the interprofessional collaborator assessment rubric ((ICAR)). Journal of interprofessional care. 2011;25(5):339–44. Epub 2011/07/08. doi: 10.3109/13561820.2011.589542 . 21732723

[34] Keshmiri F, Ponzer S, Sohrabpour A, Farahmand S, Shahi F, Bagheri-Hariri S, et al. Contextualization and validation of the interprofessional collaborator assessment rubric (ICAR) through simulation: Pilot investigation. Medical journal of the Islamic Republic of Iran. 2016;30:403. PubMed Central PMCID: PMC5038989. 27683644 PMC5038989

[35] Randita A, Widyandana W, Claramita M. IPE-COM: a pilot study on interprofessional learning design for medical and midwifery students. Journal of Multidisciplinary Healthcare. 2019:767–75. Epub 2019/10/02. doi: 10.2147/JMDH.S202522 ; PubMed Central PMCID: PMC6748318. 31571894 PMC6748318

[36] Andermo S, Forsell Ehrlich K, Forsberg Larm M, Bergström L, Alencar Siljehag P, Broberger E. Assessing students interprofessional competence using a Swedish version of the Interprofessional Collaborator Assessment Rubric. Journal of Interprofessional Care. 2023;37(4):605–12. Epub 2022/11/15. doi: 10.1080/13561820.2022.2138287 . 36373201

[37] Ozolins U, Hale S, Cheng X, Hyatt A, Schofield P. Translation and back-translation methodology in health research–a critique. Expert review of pharmacoeconomics & outcomes research. 2020;20(1):69–77. doi: 10.1080/14737167.2020.1734453 32089017

[38] McKinney W, editor Data structures for statistical computing in python. Proceedings of the 9th Python in Science Conference; 2010: Austin, TX.

[39] Creswell JW. Educational research: Planning, conducting, and evaluating quantitative and qualitative research: Pearson Education, Inc; 2012.

[40] Mahler C, Berger S, Reeves S. The Readiness for Interprofessional Learning Scale (RIPLS): A problematic evaluative scale for the interprofessional field. 2015;29(4):289–91. Epub 2015/07/16. doi: 10.3109/13561820.2015.1059652 . 26176984

[41] Lapkin S, Levett-Jones T, Gilligan C. A systematic review of the effectiveness of interprofessional education in health professional programs. Nurse education today. 2013;33(2):90–102. Epub 2011/12/27. doi: 10.1016/j.nedt.2011.11.006 . 22196075

[42] Tsakitzidis G, Van Olmen J, Van Royen P. Training in interprofessional learning and collaboration: An evaluation of the interprofessional education program in the scale-up phase in Antwerp (Belgium). Slovenian Journal of Public Health. 2021;60(3):176–81. Epub 2021/07/13. doi: 10.2478/sjph-2021-0025 ; PubMed Central PMCID: PMC8256770. 34249164 PMC8256770

[43] Zanotti R, Sartor G, Canova C. Effectiveness of interprofessional education by on-field training for medical students, with a pre-post design. BMC medical education. 2015;15:1–8. Epub 2015/07/30. doi: 10.1186/s12909-015-0409-z ; PubMed Central PMCID: PMC4518727. 26220412 PMC4518727

[44] Reilly JM, Aranda MP, Segal-Gidan F, Halle A, Han PP, Harris P, et al. Assessment of student interprofessional education (IPE) training for team-based geriatric home care: does IPE training change students’ knowledge and attitudes? Home Health Care Services Quarterly. 2014;33(4):177–93. Epub 2014/09/27. doi: 10.1080/01621424.2014.968502 . 25256717

[45] Miller R, Scherpbier N, van Amsterdam L, Guedes V, Pype P. Inter-professional education and primary care: EFPC position paper. Prim Health Care Res Dev. 2019;20:e138. Epub 2019/10/05. doi: 10.1017/S1463423619000653 ; PubMed Central PMCID: PMC6784359. 31581968 PMC6784359

[46] Carney PA, Thayer EK, Palmer R, Galper AB, Zierler B, Eiff MP. The benefits of interprofessional learning and teamwork in primary care ambulatory training settings. Journal of interprofessional education & practice. 2019;15:119–26. doi: 10.1016/j.xjep.2019.03.011

[47] Miselis HH, Zawacki S, White S, Yinusa-Nyahkoon L, Mostow C, Furlong J, et al. Interprofessional education in the clinical learning environment: a mixed-methods evaluation of a longitudinal experience in the primary care setting. Journal of Interprofessional Care. 2022;36(6):845–55. Epub 2022/02/04. doi: 10.1080/13561820.2022.2025768 . 35109762

[48] Lestari E. Community Based Interprofessional Learning Promotes Equality of Participation among Health Professions Students. Online Journal of Health and Allied Sciences [Internet]. 2018; 17(2). Available from: https://www.ojhas.org/issue66/2018-2-5.html.

[49] Alias M, Masek A, Salleh HHM. Self, peer and teacher assessments in problem based learning: Are they in agreements? Procedia-Social and Behavioral Sciences. 2015;204:309–17. doi: 10.1016/j.sbspro.2015.08.157

[50] Papinczak T, Young L, Groves M, Haynes M. An analysis of peer, self, and tutor assessment in problem-based learning tutorials. Medical teacher. 2007;29(5):e122–e32. Epub 2007/09/22. doi: 10.1080/01421590701294323 . 17885964

[51] Lew MD, Alwis W, Schmidt HG. Accuracy of students’ self‐assessment and their beliefs about its utility. Assessment & Evaluation in Higher Education. 2010;35(2):135–56. doi: 10.1080/02602930802687737

[52] Nakamura S, Itoh M, Miki Y, Kido T, Kamei H, Suzuki S, et al. Relationship between peer evaluation and interprofessional self-evaluation in a joint healthcare team-based learning class involving three universities. Fujita medical journal. 2020;6(4):102–9. doi: 10.20407/fmj.2019-017 35111530 PMC8761826

[53] Falender CA, Shafranske EP. Clinical supervision: A competency-based approach: American Psychological Association; 2004.

[54] Reeves S. Why we need interprofessional education to improve the delivery of safe and effective care. Interface-Comunicação, Saúde, Educação. 2016;20:185–97. doi: 10.1590/1807-57622014.0092

