# Supplementary material for: A study of the impact of an interprofessional education module in Vietnam on students’ readiness and competencies
Source: PLoS One. 2024 Feb 14;19(2):e0296759. doi: 10.1371/journal.pone.0296759 (PMC10866504; doi:10.1371/journal.pone.0296759)
Supplement: S1 Table — (DOCX) [file pone.0296759.s001.docx]

**S1 Table:** Learning activities of the interprofessional education module.

| **Session** | **Learning activity** |
| --- | --- |
| Session 1 | 1. Plenary session of IPE introduction 2. Getting to know each other 3. Team-building exercise 4. Debriefing |
| Session 2 | 1. Group discussion on two paper cases of medical/ethical errors, role clarification 2. Group discussion on two video-based cases of interprofessional communication and sharing leadership 3. Debriefing |
| Session 3 | 1. Communicate with a standardised patient 2. Making an interprofessional care plan 3. Consulting with a standardised patient 4. Debriefing |
| *Sessions 4 & 7* | 1. Visit a primary health facility 2. Communicate with a patient with a morbidity condition 3. Making an interprofessional care plan 4. Debriefing |
| *Sessions 5 &8* | 1. Visit a patient’s house 2. Making an interprofessional care plan for the patient and a health preventive/screaming plan for the whole family 3. Debriefing |
| *Sessions 6 & 9* | 1. Present care plans to other groups and groups of tutors from different professions 2. Debriefing 3. Self-reflection |
| *Session 10* | Organising IPCC assessment |
